# Correction: Hydrogen bonding bolstered head-to-tail ligation of functional chromophores in a 0D SbF_3_·glycine adduct for a short-wave ultraviolet nonlinear optical material

**DOI:** 10.1039/d4sc90107j

**Published:** 2024-06-12

**Authors:** Zhiyong Bai, Jihyun Lee, Chun-Li Hu, Guohong Zou, Kang Min Ok

**Affiliations:** a Department of Chemistry, Sogang University Seoul 04107 Republic of Korea kmok@sogang.ac.kr; b State Key Laboratory of Structural Chemistry, Fujian Institute of Research on the Structure of Matter, Chinese Academy of Sciences Fuzhou 350002 P. R. China; c College of Chemistry, Sichuan University Chengdu 610065 P. R. China

## Abstract

Correction for ‘Hydrogen bonding bolstered head-to-tail ligation of functional chromophores in a 0D SbF_3_·glycine adduct for a short-wave ultraviolet nonlinear optical material’ by Zhiyong Bai *et al.*, *Chem. Sci.*, 2024, **15**, 6572–6576, https://doi.org/10.1039/D4SC01353K.

An incorrect CCDC number 2049585 was originally quoted, the correct CCDC number is 2334725.

The Royal Society of Chemistry apologises for these errors and any consequent inconvenience to authors and readers.

